# Feeling Good, Living Life: Evaluation of Psychometric Properties of the Slovak Version in Children Age 8–11 Years

**DOI:** 10.1007/s10943-019-00867-x

**Published:** 2019-06-27

**Authors:** Michaela Parilakova, Peter Babincak

**Affiliations:** Institute of Psychology, Faculty of Arts, University of Presov, Ul. 17 novembra 1, Presov, Slovakia

**Keywords:** Spirituality, Spiritual well-being, Psychometric evaluation

## Abstract

The aim of this study was to psychometrically evaluate the feeling good, living life questionnaire (FGLL) in Slovak children. Specifically, we aimed to assess the internal consistency and several proofs of the measure of the construct validity. The sample consisted of 454 children in grades 3–5, age 8–11 (mean age = 9.85, 48% boys) from state (*N* = 256) and Catholic school (*N* = 198). The internal consistency of the FGLL was assessed using Cronbach’s alpha (*α*). Confirmatory factor analysis (CFA), correlation analysis (Pearson and Spearman *r*) and nonparametric Mann–Whitney *U* test were used to verify multiple proofs of the construct validity. The values of Cronbach’s alpha, apart from the scale “Relationship with Self,” show satisfactory internal consistency (*α* = 0.68–0.84). The results of the CFA support factor structure of both parts of the Slovak version of FGLL. Further proof of the construct validity was provided by significant interscale correlations. Depending on the type of school, there were no significant differences in the scale “Relationship with God.” Slovak version of FGLL is suitable for measuring spirituality or spiritual well-being of children age 8–11 years.

## Introduction

Spirituality is a multidimensional construct that cannot be strictly and clearly defined. Definitions of spirituality differ based on the dimension that is emphasized by the authors (Malinakova et al. 2017). On one hand, spirituality can be described as the “driving force” anchored in a religious belief that gives meaning to life and affinity to stability with dimensions such as the relationship with oneself, others, nature and God (Eaude 2005; Kneezel and Emmons 2006; Smith and Snell 2009), and on the other hand, spirituality can be described as a humanistic or secular phenomenon, not bound to a specific religious context (Rossiter 2010; Ryan 2006; Tacey 2000).

There are several studies (Roehlkepartain et al. 2006; Hay and Nye 2006; Ehsani et al. 2015; Tammeh et al. 2016) that confirm the importance of spirituality in human life and indicate that spirituality is associated with all aspects of health in all age groups.

The best quantitative indicator of the link between spirituality and health is the concept of spiritual well-being (Gomez and Fisher 2003; Ingersoll 1994). The National Interfaith Coalition on Aging (1975) described spiritual well-being as the affirmation of life in a relationship with oneself, community, environment and God. According to Fisher (2016), spiritual well-being reflected the quality of relationships in up to four areas, namely relationship with the oneself, relationship with others, relationship with nature and relationship with God, which are interlinked and determine the overall spiritual well-being.

With particular reference to children, Harris (2007) compares the spirituality of children to active life, where the essential element is the connection to the environment in which the child lives.

Cervantes and Arcynski (2015) argued that children’s spirituality is “an increasing awareness, subjective inner experience of wonder and curiosity, striving for something greater than oneself, faith in unseen forces and playful transcendence” (p. 246). Nye (2013) explains that “children’s spirituality is initially a natural capacity for awareness of the sacred quality of life experiences. This awareness can be conscious or subconscious, and sometimes fluctuates between both, but in both cases it can affect actions, feelings and thoughts. In childhood, spirituality is especially about being attracted toward ‘being in relation’ to other, to God, to creation or to a deeper inner sense of Self” (p. 6). Hyde (2008) and Horell’s (2004) also reported emphasis on the importance of connecting spiritually with oneself, others, the world and with God.

Currently, there are several suitable measures for measuring spirituality, e.g., spiritual well-being, but the extensive analysis of literature (Fisher et al. 2000; Fisher 2009; Moore et al. 2016) points to the fact that only a few are addressed directly to the child respondent. For instance, Stoyles et al. (2012) developed the Children’s Sensitivity Scale, which is centered on children’s ability to reflect about themselves and the world, but does not include any questions pertaining to the child’s relationship with the transcendent, oneself, others and nature. Sifers et al. (2012) used a diverse sample to develop and validate a Youth Spirituality Scale for children (7–14 years), but it is still in the stages of requiring further validation. Fisher (2004) developed the Feeling Good Living Life (FGLL) children’s spirituality measure questionnaire, which currently appears to the most appropriate way of measuring spirituality in children age 8–11.

In the Slovak Republic, there is no instrument for measuring spirituality in childhood; therefore, we decided to translate and verify the psychometric properties of the FGLL questionnaire for Slovak children. Specifically, we aimed to assess the internal consistency and several proofs of the measure of the construct validity.

## Methods

### Sample and Procedure

Our sample consisted of 454 children (48% boys) from six elementary schools, including three state (*N* = 256) and three church schools (*N* = 198). The age of the respondents ranged from 8 to 11 years (*M* = 9.85) Children attended grades 3–5, in general corresponding to age categories 8–11-year olds.

The data were collected between January and February 2017. A researcher distributed the questionnaires, while the teachers were not present in the classroom. The researcher read the questions aloud to children age 8–9, who then answered the question by indicating their response on the measure. Respondents had one class (45 min) dedicated to completing the questionnaire, with the average time for questioning being 30 min. Participation in the survey was anonymous and voluntary.

### Measures

The first step of the research was to obtain the author’s permission to translate a questionnaire into the Slovak language. Two independent Slovak native speakers translated the questionnaire. Subsequently, a single version of the questionnaire was produced from these two translations, which was translated back into the English language by a native speaker and sent for review by the author of the questionnaire. After modifications proposed by the author, in November–December 2016, the accuracy of the final version of the questionnaire was verified for the target group of 8–11-year olds with satisfactory results.

The FGLL is a questionnaire measuring spiritual well-being of children age 5–12. Its author is Fisher (2004), who designed a model, where he linked the construct of spirituality and health to the concept of spiritual well-being consisting of four domains representing the relationship to oneself (wherein one intra-relates with oneself with regards to meaning, purpose and values in life), other (as expressed in the quality and depth of inter-personal relationships, which include love, justice, hope), nature (represented in the care of the environment, in which the individual lives and associated with feelings of fear, wonder and unity with the environment), and God (relationship with something or someone beyond the human level, which involves faith, adoration and worship). Spiritual well-being reflects the quality of relationships in these four areas.

The questionnaire consists of 16 items for both parts of FGLL. Each item is answered on a 5-point Likert scale ranging from No (scored as 1) through no (2), not sure or sometimes (3), yes (4), to YES (5). The quality of the four relationships is gauged by finding the difference in overall scores between what makes a student feel good (ideal for the Feeling Good SWB-part) and their actual experience (the Living Life part). The original Cronbach’s alpha values range from .71 to 84 (Fisher 2004, 2015).

### Statistical Analyses

Firstly, descriptive analyses of the study sample were performed. Furthermore, we verified missing data and multivariate normality (skewness and kurtosis). The amount of the missing data was minimal and only five pieces of the missing data were replaced by the mean of the scale. Because the data were not multivariate normally distributed, we used the Satorra–Bentler Chi-square test method (Satorra and Bentler 1994) to correct the lack of multivariate normality.

In the second step, we calculated the internal consistency indicator Cronbach’s alpha (*α*) for each scale and the overall score of FGLL.

In the third step, we conducted confirmatory factor analyses (CFA) to assess how well the data fit the theoretical model to determine the degree of model fit, we adopted a range of criteria on goodness-of-fit statistics, namely: normed Chi-square (*χ*2/*df*) ≤ 3, a comparative fit index (CFI) ≥ 0.90, goodness-of-fit index (GFI) ≥ 0.90 a Tucker–Lewis index (TLI) ≥ 0.90, a root-mean-square error of approximation (RMSEA) ≤ 0.08 and a standardized root-mean-square residual (SRMR) ≤ 0.06 (Hu and Bentler 1998; Kline 1998; Schermelleh-Engel et al. 2003; Tabachnick and Fidell 1996). Analyses were performed using the statistical software package IBM SPSS version 23 and Mplus software version 6.12.

In the fourth step, we identified inter correlations between the scales and the overall score of FGLL using the correlation analysis. At the same time, we identified differences in the type of school using the Mann–Whitney *U* test.

## Results

The descriptive characteristics, namely the mean, standard deviation, skewness and kurtosis values are shown in Table 1.

**Table 1:** Descriptive information of FGLL

Scale total (*N* = 454)	*M*	SD	Skewness	Kurtosis
FG_S	17.30	2.47	− 1.39	2.97
FG_F	18.92	1.90	− 2.42	6.51
FG_N	15.44	3.30	− .75	.32
FG_G	17.71	3.04	− 1.84	3.69
LL_S	16.03	2.70	− .63	.27
LL_F	18.70	1.96	− 2.20	5.58
LL_N	13.76	3.36	− .23	-.12
LL_G	17.19	3.11	− 1.71	3.59
SWB_FG	65.70	7.72	− 1.28	2.27
SWB_LL	69.38	7.79	− .92	2.42

FG—part “Feeling Good”; LL—part “Living Life”; FG_S—relationship with self; FG_F—relationship with family; FG_N—relationship with nature; FG_G—relationship with god; LL-S—relationship with self; LL_F—relationship with family; LL_N—relationship with nature; LL_G—relationship with god; SWB_FG—spiritual well-being-ideal, SWB_LL—spiritual well-being experience; *M* = mean; SD = standard deviation

The test of multivariate normality (skewness, kurtosis) in several cases shows that the data were not multivariate normally distributed, since optimal skewness values range from − 1 to 1 and, in the case of kurtosis, from − 2 to 2 (George and Mallery 2010). The lack of multivariate normality can cause several problems for model testing, which include inflated Chi-square values, underestimation of fit indices and inappropriately low standard errors leading to inflated loadings and correlations (West et al. 1995). One solution of this problem is to use maximum likelihood with robust estimation. This procedure corrects for the lack of normality, resulting in a robust Chi-square statistic referred to as the Satorra–Bentler Chi-square statistic (S–B*χ*2) (West et al. 1995; Gomez and Fisher 2003; Satorra and Bentler 1994). The S–B*χ*2 as well as χ2 likelihood ratio test statistics, the closeness of fit between the unrestricted sample covariance matrix and the restricted (postulated model) covariance matrix, after correcting for multivariate non-normality (Gomez and Fisher 2003). This study used the Satorra–Bentler Chi-square statistic method.

The internal consistency of the Slovak version of the FGLL was verified by calculating the Cronbach’s alpha coefficient. The resulting values are shown in Table 2.

**Table 2:** Internal consistency of scale of FGLL (*N* = 454)

Scale	*N*	*α*
FG_S	4	.60
FG_F	4	.72
FG_N	4	.68
FG_G	4	.84
LL_S	4	.60
LL_F	4	.71
LL_N	4	.68
LL_G	4	.80
SWB_FG	16	.82
SWB_LL	16	.79

FG—part “Feeling Good”; LL—part “Living Life”; FG_S—relationship with self; FG_F—relationship with family; FG_N—relationship with nature; FG_G—relationship with god; LL-S—relationship with self; LL_F—relationship with family; LL_N—relationship with nature; LL_G—relationship with god; SWB_FG—spiritual well-being-ideal; SWB_LL—spiritual well-being experience;, α—Cronbach’s alpha; *n*—number of item

The Cronbach’s alpha coefficient can be adequate if its value is greater than .70 (EFPA 2013). In our case, Cronbach’s alpha for the scales of the “Feeling Good” part ranged from .60 to .84 and of the “Living Life” part from .60 to .80. The internal consistency of the scale “Relationship with family” and “Relationship with God” in both parts of FGLL was acceptable, i.e., Feeling Good: “Relationship with family” = .72 and “Relationship with God” = .84; Living Life: “Relationship with family” = .71 and “Relationship with God” = .80. The scale “Relationship with Nature” in both parts has gained marginal value of the internal consistency (*α* = .68) and the scale “Relationship with self” has assumed in both parts an inadequate internal consistency value (*α* = .60). The overall score of spiritual well-being in both parts of FGLL has been adequately values of internal consistency (*α* = .79–.82).

In the next step, the confirmatory factor analysis was employed to assess the suitability of the proposed factor model, comprising of four first-order factors and a single higher factor. The goodness-of-fit indices for the CFA models of FGLL are shown in Table 3.

**Table 3:** Confirmatory factor analysis fit indices of FGLL

Model	*χ*^*2*^	S–B*χ*^*2*^	*df*	*p*	S–B*χ*^*2*^*/df*	CFI	TLI	RMSEA (90% CI)	SRMR
FG_1	306.63	216.26	100	.000	2.16	.905	.886	.060	.054
FG_2	–	185.55	98	.000	1.89	.929	.913	.054	.050
LL_1	315.23	232.21	100	.000	2.32	.875	.850	.054	.058
LL_2	–	166.76	98	.000	1.70	.935	.920	.049	.045

S–B*χ*^*2*^—Satorra–Bentler Chi-Quadrat; *df*—degrees of freedom; CFI—comparative fit index; TLI—Tucker–Lewis; RMSEA—index root-mean-square error of approximation; SRMR—standardized root-mean-square residual; FG—part “Feeling Good”; LL—part “Living Life”; FG_1—original model; FG_2—modified model; LL_1—original model; LL_2 modified model

***p ˂ .001; *χ*^*2*^ Chi-Quadrat

In the case of the “Feeling Good” part, the predicted model showed parameters at the acceptability limit, as demonstrated by the inadequate value of the TLI = .886 (TLI ≥ 0.90) and the significant Chi-Quadrat (Table 3: FG_1). For this reason, we modified the original model by allowing error covariance between items 2 and 14 to covary with the third factor “Relationship with nature,” and between items 11 a 16 to covary with the fourth factor “Relationship with God.” Adequate goodness-of-fit, except for a significant Chi-Quadrat, was found for this revised model (Table 3: FG_ 2).

Similarly, in the part “Living Life,” the predicted model showed parameters at the acceptability limit, as indicated by the inadequate value of the CFI = .875 (CFI ≥ 0.90), TLI = .850 (TLI ≥ 0.90) and significant Chi-Quadrat values (Table 3: LL_1). The fit for this solution was improved by allowing error covariance between items 2 and 14 to covary with the third factor “Relationship with nature,” and between items 7 and 8 to covary with the second factor “Relationship with family.” After the inclusion of these two covariants, we obtained acceptable values of individual variables, except for a significant Chi-Quadrat (Table 3: LL_2).

**Fig. 1:**
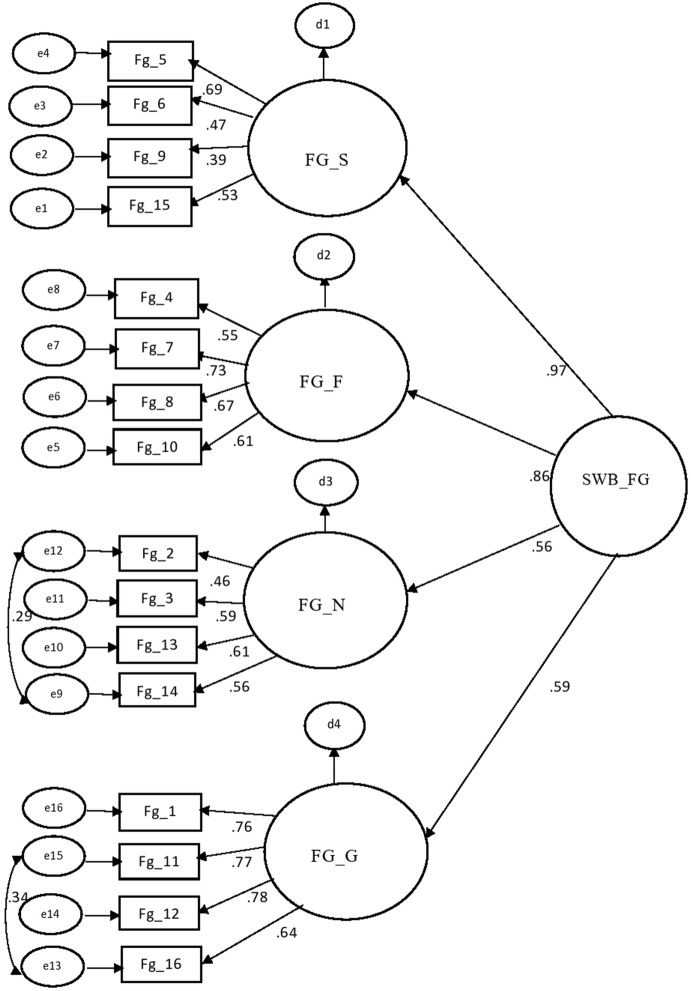
Confirmatory model of part of feeling good with standardized weights

**Fig. 2:**
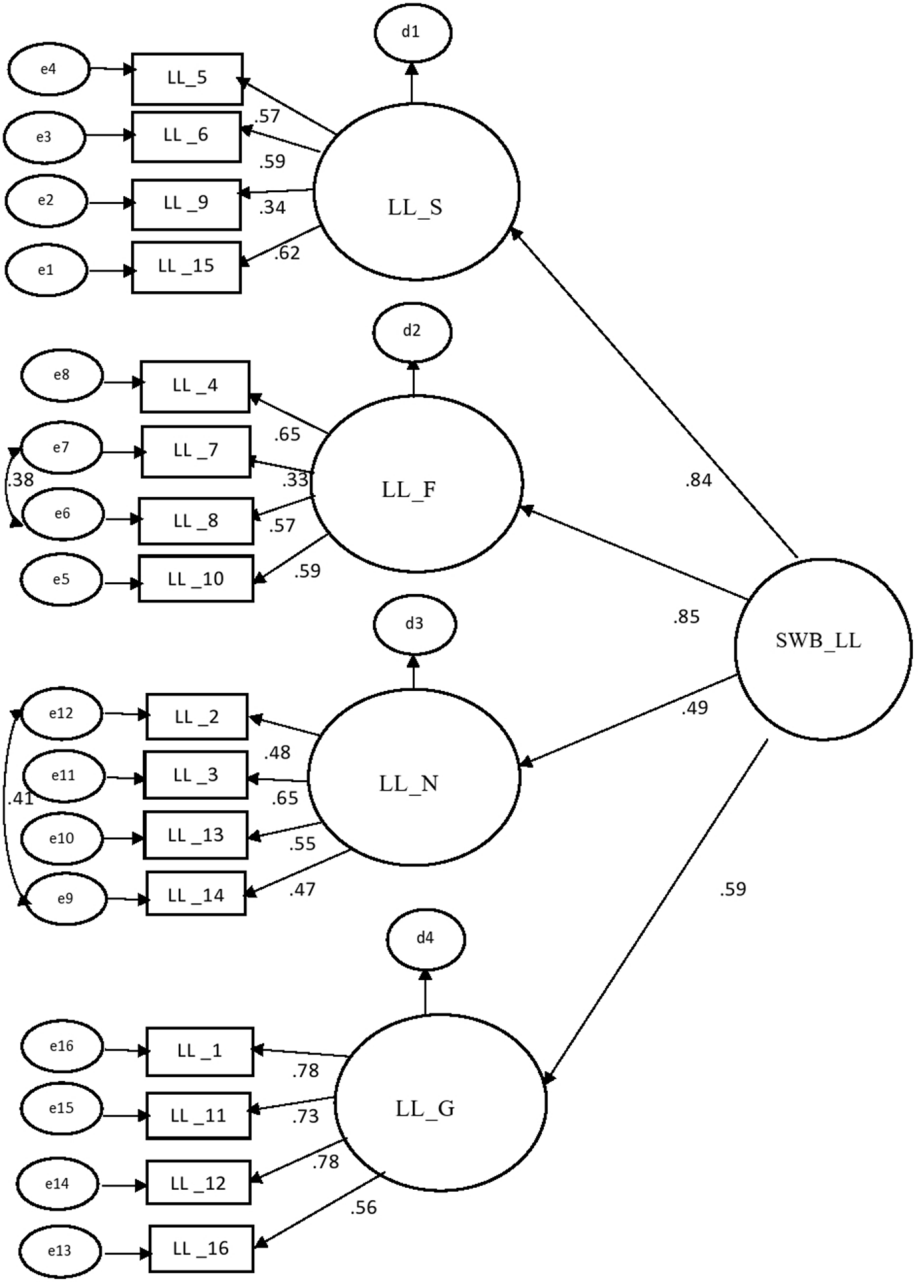
Confirmatory model of part of living life with standardized weights

The standardized factor loadings for all items were positive and significantly different from zero, ranging from .39 to .78 in the “Feeling Good” part (Fig. 1) and ranging from .33 to .78 in the “Living Life” part (Fig. 2). The result of CFA supports the factor structure of both parts of FGLL.

In the next step, we carried out the correlation analysis between the scale and overall score of FGLL.

A correlation matrix of the FGLL’s scale is presented in Table 4. As expected, each scale was significantly correlated with the overall score and with each other, where the correlation coefficient has a value of .17–.72. The results also show that individual scales correlate more strongly with their respective scale in the second part of the questionnaire than with other scales.

**Table 4:** Intercorrelations between scales of FGLL and overall score

	FG_S	FG_F	FG_N	FG_G	SWB_FG
LL_S	.64***	.35***	.30***	.28***	.48***
LL_F	.36***	.48***	.24***	.33***	.41***
LL_N	.26***	.17***	.68***	.17***	.47***
LL_G	.32***	.33***	.22***	.66***	.52***
SWB_LL	.55***	.43***	.57***	.53***	.72***

Italics marked by the Pearson correlation coefficient; ***p < .001; FG—part “Feeling Good”; LL—part “Living Life”; FG_S—relationship with self; FG_F—relationship with family; FG_N—relationship with nature; FG_G—relationship with god; LL-S—relationship with self; LL_F—relationship with family; LL_N—relationship with nature; LL_G—relationship with god; SWB_FG—spiritual well-being-ideal; SWB_LL—spiritual well-being experience; the correlation coefficient between the individual scale and its respective scale in the second part of the questionnaire is in bold

In the case of finding out the differences according to the type of school, there were no significant differences in the scale “Relationship with God.”

## Discussion

The aim of this study was to translate and verify the psychometric properties of the FGLL questionnaire for Slovak children. Specifically, we aimed to assess the internal consistency and several proofs of construct validity of measure.

There was evidence of adequate internal consistency in the scales “Relationship with family” and “Relationship with God” in both parts of FGLL, marginal internal consistency in the scale “Relationship with nature” in both parts of FGLL and in the “Relationship with self” scale, in the both parts of FGLL, there was inadequate internal consistency. The overall score of spiritual well-being in both parts of FGLL had good internal consistency. Comparing our findings with the original validation study of FGLL (Fisher 2004), we found that our results are inconsistent with the results of Fisher’s study, where values of internal consistency had good values (*α* = .71–.84) in all cases. However, we found similar results for internal consistency with the result of the second Fisher’s study (Fisher 2015), where in the “Relationship with self” scale part of Feeling Good was inadequate to Cronbach’s alpha (*α* = .57–.63). Pallant (2007) says that with short scales (of less than 10 items), it is common to find low Cronbach’s alpha values (e.g., .50), which could have been seen on the inadequate values of the Cronbach’s alpha. We also believe that the inadequate values acquired in the “Relational” scale could have been due to our poor translation of the items.

The results of CFA in the Feeling Good part show that the model had indices at the acceptability fit, namely that the TLI was below the acceptable value of .90 and the Chi-Quadrat, adjusted by the Satorra–Bentler Chi-Quadrat test, was significant. By allowing error covariance between similarly worded items, we have achieved adequate goodness-of-fit, except for a significant Chi-Quadrat. Several authors (Schlermelleh-Engel et al. 2003; Vandenberg 2006; Schumaker and Lomax 2004) claim that Chi-Quadrat is very susceptible to sample size, especially in the research sample with more than 200 respondents, where significant Chi-Quadrat is a very common phenomenon and should therefore not be used as a primary criterion for accepting or rejecting the model. Thus, it can be concluded that the factor analysis supported the factor structure of the Feeling Good part. The results of the CFA in the Living Life part show that CFI and TLI indices were below the acceptable value of .90, and Chi-Quadrat was significant. Even in this part, we have allowed error covariance between similarly worded items. After this modification, the model fit had acceptable values of individual indices expected for a significant Chi-Quadrat. In accordance with other authors (Schlermelleh-Engel et al. 2003; Vandenberg 2006; Schumaker and Lomax 2004), we do not consider Chi-Quadrat as the main indicator of the model’s rejection, so it can be said that factor analysis supported factor structure in the part Living Life too.

Construct validity also indicates significant correlations between the scales of the two parts of the FGLL questionnaire to each other and the overall score of FGLL, where the correlation coefficient ranged from .17 to .68. At the same time, the individual scales correlated more strongly with their respective scale in the second part of the questionnaire. Comparing our finding with the original validation study of FGLL (Fisher 2004), we found that our results are similar to the results of Fisher’s study. It can be stated that the individual scales measure the same construct but not the same domains, and therefore, each scale represents a well-defined area of spiritual well-being.

Our results have not demonstrated the existence of significant differences in the scale “Relationship with God” depending on the type of school. These findings contradict Fisher’s findings (2004, 2015). We believe that cultural differences have been involved in not confirming the differences, and especially the fact that in our conditions, regardless of whether it is a Catholic or State school, children are encouraged to develop religious knowledge in a similar way through the teaching of religious education. It can be said that comparing the differences in terms of type of school in our conditions does not provide adequate evidence of constructive validity of the tool.

We can summarize that our findings support the theoretical model of spiritual well-being, as proposed by Fisher (1998). They also provide evidence for the construct validity of the FGLL.

### Strengths and Limitations

This study has several important strengths that should be mentioned. Since there are no instruments in the Slovak language for measuring spirituality in childhood, this study is the first study that works with the FGLL as a tool for measuring children’s spirituality in the Slovak environment. The Slovak version of FGLL had suitable psychometric properties and could become a reference tool for studies about spirituality, spiritual well-being of young children in Slovakia. On the other hand, a limitation of our study could be that the participants were not selected by means of representative sampling, which limits the generalizability of these findings. Furthermore, our data were based on self-reports of children, which can be inaccurate or influenced by social desirability. Another limitation might be the fact that the sample was rather homogeneous with regard to religious orientation and strength of religious conviction, which also related to the non-normal distribution of the observed variables. However, despite some limitations, our research provided an image of the psychometric properties of the Slovak version of FGLL. Further research is clearly needed to further determine the reliability and validity of FGLL.

Undoubtedly, enriching would be to include a representative sample, as well as expand the sample of respondents from the secular environment and to ensure repeated measurements to verify retest reliability.

## Conclusion

Our findings suggest that the Slovak version of FGLL is suitable for measuring spirituality or spiritual well-being in childhood, which can be used with individuals and small or large groups of young children.
